# The Foundations of Literacy Development in Children at Familial Risk of Dyslexia

**DOI:** 10.1177/0956797615603702

**Published:** 2015-12

**Authors:** Charles Hulme, Hannah M. Nash, Debbie Gooch, Arne Lervåg, Margaret J. Snowling

**Affiliations:** 1 Division of Psychology and Language Sciences, University College London; 2 Department of Psychology, University of Leeds; 3 Department of Psychology, Royal Holloway College, University of London; 4 Department of Education, University of Oslo; 5 St John’s College, Oxford; 6 Department of Experimental Psychology, University of Oxford

**Keywords:** dyslexia, language impairment, reading development, reading comprehension, phonological skills, language skills, familial risk

## Abstract

The development of reading skills is underpinned by oral language abilities: Phonological skills appear to have a causal influence on the development of early word-level literacy skills, and reading-comprehension ability depends, in addition to word-level literacy skills, on broader (semantic and syntactic) language skills. Here, we report a longitudinal study of children at familial risk of dyslexia, children with preschool language difficulties, and typically developing control children. Preschool measures of oral language predicted phoneme awareness and grapheme-phoneme knowledge just before school entry, which in turn predicted word-level literacy skills shortly after school entry. Reading comprehension at 8½ years was predicted by word-level literacy skills at 5½ years and by language skills at 3½ years. These patterns of predictive relationships were similar in both typically developing children and those at risk of literacy difficulties. Our findings underline the importance of oral language skills for the development of both word-level literacy and reading comprehension.

The current study focused on early language skills and their role in predicting variations in reading skills in a large sample of children selected because they were at high risk of reading difficulties, either because of a family history of dyslexia or because they have a preschool language impairment. It is well established that in unselected samples of children, variations in reading skills are highly correlated with variations in oral language skills. In alphabetic writing systems, phonological (speech sound) skills are particularly important for learning to decode print. Indeed, two of the most important predictors of early word-reading skills across languages are phoneme awareness and letter knowledge (Caravolas et al., 2012), and there is evidence of reciprocal interaction between them (e.g., Lervåg, Bråten, & Hulme, 2009; Muter, Hulme, Snowling, & Stevenson, 2004; Perfetti, Beck, Bell, & Hughes, 1987).

The goal of reading is comprehension. In the early years of learning to read, reading comprehension depends strongly on word reading (Vellutino, Tunmer, Jaccard, & Chen, 2007): To understand print, it must first be decoded (Gough & Tunmer, 1986). However, oral language skills beyond phonology, including vocabulary knowledge and grammatical skills, are significant predictors of individual differences in reading comprehension (Muter et al., 2004). Furthermore, two recent studies have shown that improvements in oral language skills brought about by intervention translate into gains in reading comprehension (Clarke, Snowling, Truelove, & Hulme, 2010; Fricke, Bowyer-Crane, Haley, Hulme, & Snowling, 2013). In addition to being important for reading comprehension, broader oral language skills may also play a causal role in supporting the development of reading-accuracy skills. For example, vocabulary knowledge appears to be particularly important in English for learning to read irregular words that cannot be decoded phonologically (e.g., Ricketts, Nation, & Bishop, 2007).

Three large-scale studies have investigated the impact of early oral language skills on later literacy skills in the transition from preschool to formal schooling. Storch and Whitehurst (2002) found that oral language skills did not affect reading development until Grade 3 when their contribution was to reading comprehension; before that stage, reading comprehension was highly dependent on decoding skills, as measured by reading accuracy. In contrast, the National Institute of Child Health and Human Development (NICHD) Early Child Care Research Network (2005) found that there was a direct effect of oral language on reading-accuracy skills in Grade 1 as well as on reading comprehension in Grade 3. Finally, a recent longitudinal study of twins (Christopher et al., 2015) assessed a broad range of oral language skills prior to reading instruction. This study demonstrated that variations in vocabulary and verbal memory, as well as prereaders’ print knowledge, rapid naming ability, and phonological awareness, were important predictors of later reading and spelling accuracy, and furthermore, these longitudinal influences appeared to reflect both genetic and environmental influences on development.

Together, these findings from groups of typically developing children highlight the important contribution of oral language skills to reading development, though only the study of Christopher et al. (2015) included measures of rapid automatized naming (RAN), which is known to be a powerful predictor of the development of word-reading skills (Caravolas, Lervåg, Defior, Seidlová Málková, & Hulme, 2013; Caravolas et al., 2012; Lervåg et al., 2009). Our particular focus in the present research, however, was on children selected to be at risk of later reading difficulties. Three prospective longitudinal studies of children at familial risk of dyslexia have attempted to identify factors predisposing children to later difficulties with literacy and preliteracy skills. Snowling, Gallagher, and Frith (2003) assessed the impact of early language skills on later reading skills (assessed at 8 years) in an English family-risk sample. Language together with letter knowledge assessed at 3 years 9 months predicted phonological awareness at the age of 6 years, which, in turn, together with grapheme-phoneme knowledge, predicted word-level reading skills at 8 years. Torppa, Lyytinen, Erskine, Eklund, and Lyytinen (2010), using data from a large Finnish family-risk study, showed that receptive language at 2½ years predicted phonological awareness (and performance on a RAN objects task) at 3½ years, whereas expressive language at 2½ years predicted letter knowledge a year later. Between 3½ and 5½ years, oral language skills continued to be important, with expressive language predicting literacy-related measures (phonological awareness, letter naming, and RAN) at 5½ years. Reading outcomes in Grade 3 (reading accuracy and fluency) were predicted by phonological awareness, letter naming, and RAN at 5½ years. Finally, Carroll, Mundy, and Cunningham (2014) reported a family-risk study starting later when children were between the ages of 4 years 5 months and 7 years; they found that language and phonological-processing scores in the early school years were predictors of later variations in reading and spelling skills across the whole sample.

In the present study, we aimed to extend the findings of these three family-risk studies by investigating the role of early oral language and speech skills as influences on reading outcomes approximately 1 and 3 years after the introduction of formal reading instruction. Given the importance of oral language as a predictor of later phonological awareness and letter knowledge, we also included children with preschool-specific language impairment to increase the range of language skills represented in our sample. Hayiou-Thomas et al. (2006) used data from a twin study to argue for two separable language factors: a general language factor with loadings from measures tapping syntax and semantics and an “articulation” factor with loadings from measures tapping phonological skills (articulation and nonword repetition).

In the analyses presented here, we assessed the relative impact of speech skills (assessed by measures of articulation and word and nonword repetition—similar to the articulation measure of Hayiou-Thomas et al., 2006) compared with broader language skills (vocabulary and grammatical skills) on reading development in this large and diverse sample of children. Our particular interest was to identify whether early speech and language skills predict variations in preliteracy skills (particularly phoneme awareness, letter knowledge, and RAN) and thereby influence literacy skills. A further question was whether children at high risk of reading difficulties would show a similar pattern of relationships between early language and later literacy skills as typically developing children.

## Method

### Design

This was a prospective longitudinal study of children at risk of reading difficulties either because of a family history of dyslexia or because of preschool language difficulties (the latter drawn from a sample of children receiving speech and language therapy at the beginning of the study). Data were collected in five assessments conducted at approximately yearly intervals. Data from two assessments before the beginning of formal schooling (Time 1, Time 2) and two after school entry (Time 3, Time 5) are reported here. Time 1 was when the children were 3 to 4 years old, Time 2 occurred at 4 to 5 years, Time 3 at 5 to 6 years, and Time 5 at 7 to 9 years (mean age at Time 5 = 8.7 years). Data were also collected at Time 4 (6 to 7 years) but are not reported here. The sample size was determined largely by the practicalities of participant recruitment: We sought to recruit as many children at familial risk of dyslexia and children with preschool language difficulties (within a narrow age range) as possible in the area where the study was conducted (York, United Kingdom).

### Participants

Ethical clearance for the study was provided by the University of York Department of Psychology Ethics Committee and the National Health Service Research Ethics Committee. Families were recruited using advertisements and speech- and language-therapy services. Our exclusion criteria consisted of being a monozygotic twin, having a chronic illness, being deaf, speaking English as a second language, being cared for by a local authority, and having a known neurological disorder, such as cerebral palsy, epilepsy, or autism spectrum disorder. Of the 245 children recruited, none met these criteria at Time 1. Parents provided informed consent for their family’s involvement.

Following recruitment, children were classified using a two-stage process determining whether they were at familial risk of dyslexia and then using diagnostic criteria to determine whether they had a specific language impairment. This led to the classification of children into four groups according to family and language status: typically developing (*n* = 71), familial risk of dyslexia only (*n* = 86), language impaired only (*n* = 36), and familial risk of dyslexia with language impairment (*n* = 37). In addition, 15 children had been referred as having a specific language impairment but did not fulfil our research criteria for the language-impaired group (see Nash, Hulme, Gooch, & Snowling, 2013, for further details). Another 15 children entered the project at the second time point (5 typically developing, 7 familial risk only, 1 familial risk with language impairment, 1 language impaired only, and 1 referred as language impaired but who did not meet diagnostic criteria). There was a small amount of attrition between time points, which was greatest between Time 1 and Time 2 (*n* = 16) and reduced between later assessments (Time 2 to Time 3: *n* = 3, Time 3 to Time 4: *n* = 2, Time 4 to Time 5: *n* = 5).

### Tests and procedures

Cognitive, language, and literacy tests were administered at each time point. Research assistants were trained and observed by the project manager to ensure fidelity, and when possible, the same assistant visited the child on each occasion. We report details of the measures used only in the present analyses. At Times 1 and 2, the assessments took place at home, in a single 1½-hr session at Time 1 and across two 1-hr sessions at Time 2, with breaks as necessary. At Times 3 and 5, the assessments usually took place at school and lasted for approximately 2 hr with a break. The tasks were administered in a fixed order.

#### Tasks administered at Time 1

We used seven measures of speech and language at Time 1. The articulation subtest of the Diagnostic Evaluation of Articulation and Phonology (Dodd, Hua, Crosbie, Holm, & Ozanne, 2002) provided a measure of the percentage of consonants correctly produced. Children named 30 pictures (e.g., pig, moon, sheep, five, television); if they could not produce the name spontaneously, the examiner spoke it and asked the child to repeat it.

The Preschool Repetition subtest from the Early Repetition Battery (Seeff-Gabriel, Chiat, & Roy, 2008) was used to assess word- and nonword-repetition ability as well as sentence-repetition ability. For word- and nonword-repetition ability, children were asked to repeat 18 words and 18 nonwords (6 one-syllable, 6 two-syllable, and 6 three-syllable words and nonwords). Nonwords were created from the words by altering the vowel in the one-syllable items and swapping two consonants in the multisyllabic items (e.g., *lamb* → “lom,” *machine* → “shameen,” *dinosaur* → “sinodaur”). For sentence repetition, children repeated 16 sentences increasing in length and complexity (e.g., a fairly simple sentence would be “The cat ate a big mouse”). The total number of sentences, content words, function words, and grammatical inflections repeated correctly was recorded.

The Clinical Evaluation of Language Fundamentals—Preschool (2nd UK edition; Wiig, Secord, & Semel, 2006) was used to assess basic concepts, expressive vocabulary, and sentence structure. For basic concepts, children heard a sentence (e.g., “Point to the one that is long”) and were shown three pictures. They had to select the picture that represented the concept. Expressive vocabulary was assessed by asking the child to name pictured objects (e.g., carrot, telescope) or to describe what a person in a picture was doing (e.g., riding a bike). For sentence structure, the child heard a sentence (e.g., “The bear is in the wagon”) and had to select the picture that conveyed its meaning from among four possibilities. The sentences included a range of different syntactic structures.

#### Tasks administered at Time 2

At Time 2, just at or just before the onset of formal literacy instruction, grapheme-phoneme knowledge was defined by measures of letter-sound knowledge and of writing letters to dictation, and phoneme awareness was defined by tests involving isolation of initial and final phonemes in spoken nonwords.

The York Assessment of Reading for Comprehension (YARC; Snowling et al., 2009) was used to assess letter-sound knowledge. Children were shown 32 single letters and digraphs one at a time and asked what sound each one made. If they provided the letter name, they were prompted to provide the sound. We also administered a letter-writing task in which we asked the child to write 10 letter sounds, 5 of which had consistent sound-to-letter mappings (/b/, /h/, /m/, /g/, and /w/) and 5 of which had inconsistent sound-to-letter mappings (/s/, /k/, /ʤ/, /f/, /z/). One point was awarded for each letter written correctly (for the inconsistent items, either letter was accepted).

Phoneme awareness was measured for the beginnings and endings of words. For phoneme isolation of initial sounds, the child was asked to repeat a spoken nonword and then to say its first sound. There were two demonstration and two practice items followed by eight monosyllabic test items (four CVC and four CCVC; C = consonant, V = vowel). Testing was discontinued after four incorrect responses. Following the initial isolation task, the child was asked to say the last sound in each nonword. There were two practice and eight test items, four CVC and four CVCC. Testing was discontinued after four incorrect responses.

We conducted two versions of the RAN task: colors and objects. Children were first asked to name each of the 5 stimuli (objects: pictures of a dog, eye, key, lion, and table; colors: squares colored brown, blue, black, red, and green) to check that they knew the names. Following this, children were presented with an 8 × 5 array of stimuli (each of the 5 stimuli was presented eight times in a random order) and were told to name each of the stimuli (moving from left to right) as quickly as possible. The time taken to name all 40 stimuli and the number of errors made were recorded. RAN rate was calculated as the number of correct responses (maximum of 40) divided by time (in seconds).

#### Tasks administered at Times 3 through 5

We measured word-level literacy skills at Times 3 and 4 and reading comprehension at Time 5. Early word reading was assessed at Times 3 and 4 using the YARC. The child read aloud 30 single words, graded in difficulty. Half of the words were phonemically regular (decodable), and the other half were irregular. Each correct response scored 1 point; testing was discontinued if the child made 10 consecutive reading errors. Single-word reading ability was assessed at Times 3, 4, and 5 with the Single Word Reading Test (Foster, 2007). Children read 60 words of increasing difficulty. Testing was discontinued after five consecutive errors or refusals to read the word. Spelling ability was assessed at Times 3 and 4 by asking children to spell five words (dog, cup, tent, book, heart), each represented by a picture. They first named each picture, but if they could not or if they made an error, the examiner provided the name before the child attempted to write the word.

Finally, reading comprehension was assessed at Time 5 with the YARC (Snowling et al., 2009). The Passage Reading subtest of the YARC required the child to read a series of short texts during which reading errors were corrected by the examiner up to a given number, at which point testing was discontinued following procedures in the test manual. The passage the child started with was determined by his or her single-word reading-accuracy level. For each passage that was not discontinued because of reading errors, the child then answered eight spoken comprehension questions. Accuracy, reading rate, and comprehension-ability scores were calculated based on the two most difficult passages the child read. Reading comprehension was measured using ability scores.

## Results

We wished to assess the patterns of predictive relationships between early measures of language and speech skills and later literacy skills in children at risk of literacy difficulties and in control children. Initial explorations of the data indicated that the levels of performance and pattern of relationships between variables were very similar in the children at familial risk of dyslexia and those referred because of concerns about preschool language difficulties. Children who met one or both of these criteria were therefore combined for the purposes of further analyses (this group will be referred to as the *at-risk group* in what follows).

The means, standard deviations, and reliabilities for all variables for the at-risk and typically developing control groups are shown in Table 1. As expected, the control group performed better than the at-risk group on all the language and literacy measures, with moderate to large effect sizes. At Time 1, the language measures and speech measures were moderately correlated in the at-risk group; however, the correlations between these measures were lower in the control group because of restrictions of range on some measures (see Table 2).

**Table 1.** Performance of the Samples on Key Language and Literacy Measures

	At-risk group			Control group				
Time and measure	*n*	*M*	*SD*	*n*	*M*	*SD*	Reliability (α)	Cohen’s *d*
Time 1								
Age in months	174	45.13	3.65	71	44.69	3.20	—	−0.12 [–0.40, 0.15]
Articulation (100)	172	73.60	20.43	71	89.48	7.58	—	0.90 [0.61, 1.18]
Word repetition (18)	161	13.51	3.96	68	16.74	1.93	.89	0.93 [0.63, 1.22]
Nonword repetition (18)	160	10.65	3.94	67	14.12	2.52	.89	0.97 [0.67, 1.27]
Basic concepts (18)	171	13.39	3.54	71	16.35	1.64	—	0.95 [0.66, 1.24]
Vocabulary (40)	169	15.00	7.06	71	20.69	5.24	.82	0.87 [0.58, 1.15]
Sentence structure (22)	170	11.21	4.16	71	14.39	3.27	.78	0.81 [0.52, 1.10]
Sentence repetition (16)	144	4.17	4.23	67	9.27	4.28	—	1.18 [0.87, 1.49]
Time 2								
Letter-sound knowledge (32)	169	15.03	10.03	74	19.78	9.36	.95	0.48 [0.21, 0.76]
Writing letters (10)	169	3.30	3.03	74	4.65	3.05	.85	0.44 [0.17, 0.72]
Phoneme isolation: beginning of words (8)	126	4.59	2.95	70	5.76	2.54	.91	0.42 [0.12, 0.71]
Phoneme isolation: end of words (8)	121	2.06	2.87	68	3.44	3.32	.95	0.45 [0.15, 0.75]
RAN objects rate	150	.65	.19	71	.79	.17	—	0.78 [0.49, 1.07]
RAN colors rate	137	.56	.18	67	.69	.19	—	0.72 [0.42, 1.02]
Time 3								
Early word reading (30)	167	13.89	8.70	74	20.15	8.04	.98	0.74 [0.45, 1.01]
Single-word reading (60)	165	7.88	9.30	74	15.52	13.53	—	0.71 [0.43, 0.99]
Spelling (5)	167	1.66	1.34	74	2.55	1.22	—	0.68 [0.40, 0.96]
Time 5								
Reading comprehension (88)	155	54.66	9.35	72	60.58	8.71	.77	0.65 [0.36, 0.93]

Note: For most measures, the maximum possible score is given in parentheses. For rapid automatized naming (RAN), the rate was calculated as the number of correct responses (maximum of 40) divided by time (in seconds). Cohen’s *d*s for the mean difference between groups are adjusted for unequal sample size (Rosnow, Rosenthal, & Rubin, 2000); values in brackets are 95% confidence intervals.

**Table 2.** Correlations Between Measures for the Control Group (Above the Diagonal) and the At-Risk Group (Below the Diagonal)

Variable	1	2	3	4	5	6	7	8	9	10	11	12	13	14	15	16	17	18	19	20	21
1. Age at Time 1	—	.25	.19	.19	−.03	−.07	.17	.08	.32	.21	.21	.32	.04	.02	.01	−.06	.11	−.07	−.08	−.05	.14
2. Articulation at Time 1	.22	—	.25	−.08	−.06	−.04	.10	.06	.02	.07	.17	.12	.13	.06	−.03	−.11	.02	−.09	−.07	−.06	.05
3. Basic concepts at Time 1	.27	.24	—	.34	.48	.22	.24	.35	.32	.32	.38	.22	.40	.24	.27	−.04	.19	.13	.26	.27	.37
4. Vocabulary at Time 1	.28	.27	.61	—	.28	.05	.24	.27	.10	.08	.22	.07	.19	.14	.07	−.08	.01	−.03	.09	−.08	.44
5. Sentence structure at Time 1	.21	.25	.63	.55	—	.21	.04	.26	.18	.16	.04	.15	.09	.27	.15	.10	.09	.07	.11	.13	.37
6. Word repetition at Time 1	.24	.72	.41	.41	.34	—	.30	.32	.04	.14	.07	.13	.21	.28	.12	−.08	.06	.03	.07	.09	−.02
7. Nonword repetition at Time 1	.28	.58	.34	.38	.32	.72	—	.42	.06	.14	.32	.15	.24	.16	.08	−.16	.04	−.15	.05	−.04	.14
8. Sentence repetition at Time 1	.32	.35	.57	.55	.56	.51	.54	—	.08	.15	.36	.16	.05	.03	.17	.02	.23	.02	.10	.04	.24
9. Letter-sound knowledge at Time 2	.42	.26	.32	.26	.30	.27	.20	.38	—	.69	.49	.58	.31	.30	.55	.43	.48	.42	.46	.41	.21
10. Writing letters at Time 2	.40	.30	.35	.27	.32	.32	.29	.43	.83	—	.49	.61	.34	.36	.57	.33	.53	.38	.42	.43	.16
11. Phoneme isolation: beginnings at Time 2	.17	.19	.38	.32	.39	.35	.29	.42	.51	.48	—	.56	.26	.22	.33	.09	.41	.15	.23	.11	.09
12. Phoneme isolation: endings at Time 2	.15	.16	.31	.20	.38	.17	.19	.45	.52	.52	.62	—	.24	.31	.47	.51	.44	.27	.33	.20	.05
13. RAN objects rate at Time 2	.13	.12	.42	.20	.29	.08	.05	.29	.37	.35	.34	.35	—	.70	.26	.15	.31	.19	.28	.22	.21
14. RAN colors rate at Time 2	.12	.15	.17	.00	.14	.16	.04	.19	.34	.29	.28	.26	.60	—	.30	.19	.32	.20	.23	.23	.09
15. Early word reading at Time 3	.28	.23	.41	.36	.34	.33	.38	.40	.61	.61	.52	.53	.41	.35	—	.58	.70	.73	.78	.66	.21
16. Single-word reading at Time 3	.27	.25	.38	.30	.35	.27	.33	.39	.55	.56	.43	.52	.34	.28	.90	—	.55	.39	.44	.46	−.03
17. Spelling at Time 3	.25	.30	.47	.35	.38	.35	.34	.39	.51	.56	.41	.41	.39	.31	.79	.77	—	.62	.64	.58	.11
18. Early word reading at Time 4	.14	.15	.29	.22	.16	.20	.27	.30	.48	.50	.41	.42	.30	.27	.77	.63	.67	—	.78	.55	.21
19. Single-word reading at Time 4	.19	.19	.35	.26	.26	.24	.35	.39	.50	.54	.46	.49	.35	.29	.87	.85	.74	.84	—	.72	.33
20. Spelling at Time 4	.15	.18	.30	.14	.23	.19	.29	.33	.54	.60	.41	.49	.32	.30	.81	.77	.75	.78	.86	—	.10
21. Reading comprehension at Time 5	.14	.17	.37	.45	.31	.26	.21	.38	.32	.27	.43	.30	.22	.10	.42	.38	.41	.47	.45	.34	—

Note: RAN = rapid automatized naming.

The Time 2 letter-knowledge variables were strongly intercorrelated and moderately correlated with phoneme measures. Time 2 measures of letter knowledge and phoneme awareness correlated moderately with reading and spelling at later time points. The two RAN measures were strongly correlated with each other and moderately to weakly correlated with the other tests. At Time 3, word-level literacy skills were measured by two word-reading tests and one spelling test, which were all strongly intercorrelated.

**Fig. 1.**
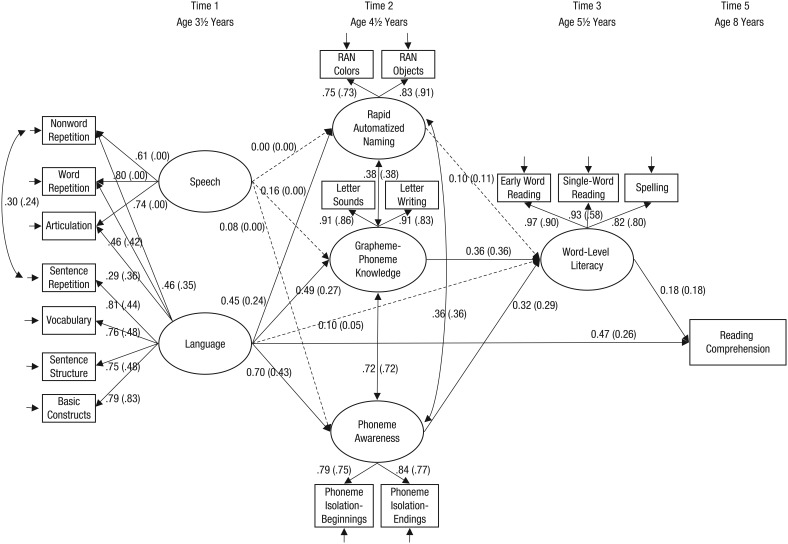
Two-group structural equation path model showing the longitudinal relations between Time 1 (preschool) measures of language and speech, Time 2 preliteracy skills, Time 3 word-level literacy skills, and Time 5 reading comprehension for the at-risk and control samples. Ellipses indicate latent variables, and rectangles indicate observed variables. On each path, values for the at-risk group are outside parentheses, and values for the typically developing (control) group are inside parentheses. Values on single-headed arrows from the latent to the observed variables are standardized factor loadings (or standardized regression weights for reading comprehension), and values on single-headed arrows between the latent variables are standardized regression weights. Double-headed arrows indicate correlations (covariances). Solid lines indicate statistically significant relationships, and dashed lines indicate statistically nonsignificant relationships.

Our principal interest was to trace possible causal influences from early variations in language and speech skills to variations in later preliteracy and literacy skills. For this purpose, a two-group structural equation model was constructed (see Fig. 1) using Mplus (Version 7.31; Muthén & Muthén, 2015) with missing data handled using full-information maximum-likelihood estimation. Before creating the two-group structural equation model, we established that strong (scalar) measurement invariance was present for all latent variables in the model because constraining the unstandardized factor loadings and intercepts to be equal across groups resulted in no significant change in fit, Δχ^2^(23) = 30.464, *p* = .137.

We wished to assess the possibly separable influences of language and speech skills on later literacy skills. In the model in Figure 1, all seven measures of language and speech were used to define a language factor, while a speech factor was defined by the three speech measures alone (articulation, word repetition, nonword repetition). The language and speech factors in this model were fixed to be uncorrelated. Therefore, the speech factor in this model reflects the variance in the three speech measures that is independent of the language factor. This allowed us to detect any influence that speech alone might have on later constructs independent of the influence of broader language skills.

For the full structural model, we first tested whether the regressions, covariances, and residual variances of the latent variables differed between groups. As this was not the case, Δχ^2^(21) = 21.538, *p* = .427, we fixed them to be equal for both samples. Figure 1 shows standardized path weights for both groups (these coefficients differ slightly between groups because of differences in variance between the groups). In this model, the variance of the latent speech variable was fixed to zero in the typically developing sample because the estimated variance was negative (−.009) but nonsignificant (*p* = .958). Accordingly, all factor loadings and regressions were fixed to zero in this group.

At Time 1, the structural model consisted of two independent latent variables (speech and language). Language at Time 1 predicted variations in the three latent variables at Time 2: grapheme-phoneme knowledge, phoneme awareness, and RAN. In contrast, variations in speech skills at Time 1 did not predict variations in these Time 2 measures (after language skills had been controlled). The three latent variables at Time 2 were moderately to strongly correlated with each other (*r*s = .36–.72), and two of these (grapheme-phoneme knowledge and phoneme awareness, but not RAN) predicted Time 3 word-level literacy skills. Finally, reading comprehension at Time 5 was predicted by both language at Time 1 and word-level literacy skills at Time 3. In addition to these direct effects, we found significant indirect effects of language at Time 1 (through the Time 2 constructs) on Time 3 word-level literacy skills for both the at-risk group (β = 0.45, *p* = .000) and the control group (β = 0.25, *p* = .000). Significant indirect effects of language at Time 1 were also found on reading comprehension at Time 5 (through the Time 2 and 3 constructs) for both the at-risk group (β = 0.08, *p* = .030) and the control group (β = 0.04, *p* = .030).

Overall, this model accounted for 60% of the variance in word-level literacy skills at Time 3 and 34% of the variance in reading comprehension at Time 5 in the at-risk sample (47% and 12%, respectively, for the control group). The model fitted the data very well, χ^2^(247) = 289.48, *p* = .033, root-mean-square error of approximation = .036 (90% confidence interval = [.011, .053]), comparative fit index = .98, Tucker-Lewis index = .98, which confirms that the structure of the underlying abilities specified in the measurement model fitted the data well.

## Discussion

This study followed the development of a large sample of children who were at risk of reading problems either because they had a family history of dyslexia or because they had preschool language difficulties, together with a group of typically developing control children. At Time 1, we found that children’s performance on the language measures could be described by two latent factors. A broad language factor with loadings from measures of both phonological (speech) and nonphonological language skills, and an independent speech factor that accounted for those aspects of speech that are independent of broader language skills (likely to reflect speech-motor processes). Only the language factor predicted later variations in phoneme awareness, grapheme-phoneme knowledge, and RAN at Time 2. In turn, phoneme awareness and grapheme-phoneme knowledge at Time 2 predicted variations in word-level literacy skills at Time 3. Finally, word-level literacy skills at Time 3 and language at Time 1 accounted for substantial proportions of the variance in reading comprehension at Time 5.

The finding that language skills could be described by two latent factors corroborates the findings of Hayiou-Thomas et al. (2006) and supports the idea that language and speech skills tap partially separate abilities. Our finding that only the language factor predicted variations in preliteracy skills at Time 2, which subsequently predicted word-level literacy, is consistent with findings from the family-risk study of Snowling et al. (2003; see also Hindson et al., 2005). Our findings are also in line with studies of “late talkers” who show broad deficits on a range of literacy and language measures when assessed at school age (e.g., Preston et al., 2010).

The finding that grapheme-phoneme knowledge and phoneme awareness together predict word-level decoding skills is in line with a large body of evidence (see Melby-Lervåg, Lyster, & Hulme, 2012). Moreover, their role in mediating the impact of oral language skills on early word-level literacy skills is consistent with findings from other studies of children at high risk of dyslexia in whom early language delay is a characteristic feature (e.g., Scarborough, 1990; Snowling et al., 2003; Torppa et al., 2010). In the current study, RAN did not predict variations in word-level literacy skills, which may reflect the fact that RAN appears to predict variations in word-reading skills more strongly at later ages (see Caravolas et al., 2013).

In line with the simple view of reading (Gough & Tunmer, 1986) and with previous longitudinal findings (NICHD Early Child Care Research Network, 2005; Muter et al., 2004; Storch & Whitehurst, 2002), our results showed that reading comprehension builds on word-reading accuracy but is also heavily influenced by variations in oral language skills. In the current study, it is striking that oral language skills assessed at 3½ years had a direct influence on the development of reading comprehension measured at age 8½ years. We believe it is likely that the effects of oral language skills on reading comprehension are causal, since training studies indicate that interventions to boost children’s oral language-comprehension skills also improve reading-comprehension skills (Clarke et al., 2010; Fricke et al., 2013).

This study is one of a few (e.g., Carroll et al., 2014) that have examined reading outcomes in children selected either as being at familial risk of dyslexia or with a preschool language impairment. Given that poor phonological skill is a major risk factor for poor reading, and that the predictive relationships between phonology and reading were the same in the children at familial risk and those with language impairment, we combined these groups for the purposes of longitudinal analyses into one at-risk group. It is clear that, on average, the at-risk group showed substantial deficits in word-level literacy skills at Time 3 (after roughly 1 year of formal schooling). Being at familial risk of dyslexia, or being referred for preschool language difficulties, are both associated with substantially worse reading outcomes after 1 year in school. A striking finding is that the predictors of early reading outcome are essentially identical in both groups and essentially the same as in typically developing children. We believe this pattern of results supports the idea that early cognitive risk factors for later reading difficulties (early language problems and later problems with phoneme awareness and learning letter-sound relationships) are best thought of as representing continuous risks with an approximately normal distribution in the population (see also Fletcher et al., 1994).

In summary, the findings from the present study make it clear that the development of reading depends critically on oral language skills. Children at familial risk of dyslexia show broad deficits in oral language skills in the preschool years, and a proportion of these children satisfy the criteria for the diagnosis of a language impairment. Poor oral language skills in turn appear to compromise the later development of decoding (via problems in acquiring letter-sound knowledge and phoneme awareness) as well as reading-comprehension abilities. It follows that education in the early years should focus not only on phonological (speech sound) and phonic (understanding letter-sound relationships) skills but also on the development of the broader language skills that provide the foundation both for learning to decode print and for the subsequent development of reading comprehension.
